# Heat‐killed *Mycolicibacterium aurum* Aogashima: An environmental nonpathogenic actinobacteria under development as a safe novel food ingredient

**DOI:** 10.1002/fsn3.2413

**Published:** 2021-07-21

**Authors:** Imen Nouioui, Timothy Dye

**Affiliations:** ^1^ Devonshire Building Newcastle University School of Natural and Environmental Sciences Newcastle Upon Tyne United Kingdom of Great Britain and Northern Ireland; ^2^ Aurum Switzerland AG Zurich Switzerland

**Keywords:** DSM 33539, *Mycolicibacterium aurum* Aogashima, novel food, safety

## Abstract

Over the last few decades, a wealth of evidence has formed the basis for “the Old Friends hypothesis” suggesting that, in contrast to the past, increasingly people are living in environments with limited and less diverse microbial exposure, with potential consequences for their health. Hence, including safe live or heat‐killed microbes in the diet may be beneficial in promoting and maintaining human health. In order to assess the safety of microbes beyond the current use of standardized cultures and probiotic supplements, new approaches are being developed. Here, we present evidence for the safety of heat‐killed *Mycolicibacterium aurum* Aogashima as a novel food, utilizing the decision tree approach developed by Pariza and colleagues (2015). We provide evidence that the genome of *M. aurum* Aogashima is free of (1) genetic elements associated with pathogenicity or toxigenicity, (2) transferable antibiotic resistance gene DNA, and (3) genes coding for antibiotics used in human or veterinary medicine. Moreover, a 90‐day oral toxicity study in rats showed that (4) the no observed adverse effect level (NOAEL) was the highest concentration tested, namely 2000 *μ*g/kg BW/day. We conclude that oral consumption of heat‐killed *M*. *aurum* Aogashima is safe and warrants further evaluation as a novel food ingredient.

## Graphical abstract

blurb for etocThere is growing realization of the health benefits of restoring human exposure to a more diverse range of microbes than we experience in increasingly urbanized lifestyles. In addition to probiotics, other environmental nonpathogenic organisms, such as environmental saprophytic nontuberculous (NTB) mycobacteria species, were once commonly present in the human diet. Here, we present evidence for the safety of heat‐killed *Mycolicibacterium aurum* Aogashima as a novel food, utilizing the decision tree approach developed by Pariza and colleagues (2015).

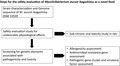

## 1 INTRODUCTION

The interaction between the human host and nonpathogenic ubiquitous environmental microorganisms, present throughout human evolution, recently emerged as an area of scientific interest and has evolved into “the Old Friends hypothesis” (Flandroy et al., 2018; Lowry et al., 2016; Rook et al., 2004, 2013). This awareness has reached consumers alike, who are increasingly willing to adjust their dietary habits to achieve improved well‐being (Marco et al., 2020). Indeed, intake of specific food and food supplements is one way to modulate exposure to what have been broadly considered “good” bacteria. The long list of “healthy” foods containing such bacteria includes fermented dairy products like yogurt and kefir, as well as fermented foods such as miso, kimchi, and sauerkraut and beverages such as kombucha tea. Members of the genera *Bacillus*, *Bifidobacterium, Enterococcus*, *Lactobacillus*, *Saccharomyces,* and *Streptococcus* are most commonly found in these foods, and together they are known as “probiotics” (Di Cerbo et al., 2016; Hori et al., 2020). According to the revised definition of the Food and Agriculture Organization (FAO)/World Health Organization (WHO), as well as in the public perception, probiotics are nonpathogenic live microorganisms that, when administered in adequate amounts, confer a health benefit to the host, such as improvement in metabolism and intestinal flora and modulation of immune functions (Aponte et al., 2020; FAO & WHO, 2002; Hill et al., 2014; Wilkins & Sequoia, 2017). The probiotic market is growing rapidly, buoyed by both foods and supplements intended to enhance wellness in healthy individuals, and by preparations for the dietary management of diseases (Grumet et al., 2020).

In addition to probiotics, other environmental nonpathogenic organisms are, or were at some point, commonly present in the human diet, such as environmental saprophytic nontuberculous (NTB) mycobacteria species. Based on recent comparative genomic studies, the genus *Mycobacterium* (Lehmann, 1896) was divided into an emended genus *Mycobacterium*, to which pathogenic species belong, and four novel genera: *Mycolicibacter* (type species: *Mycolicibacter* terrae), *Mycolicibacillus* (type species: *Mycolicibacillus trivialis*), *Mycobacteroides* (type species: *Mycobacteroides abscessus*), and *Mycolicibacterium* (type species: *Mycolicibacterium fortuitum*) (Gupta et al., 2018). The genus *Mycolicibacterium* encompasses rapidly growing NTB species, many of which have routinely been isolated from municipal water supplies (Falkinham et al., 2001; Falkinham, 2009; Fernandez‐Rendon et al., 2012; Imwidthaya et al., 1989; Kubalek & Mysak, 1996; Le Dantec et al., 2002a, 2002b; Martın et al., 2000; Moghim et al., 2012; Nasr‐Esfahani et al., 2012; Pontiroli et al., 2013; Scarlata et al., 1985; Vaerewijck et al., 2005). NTB mycobacteria, which include the mycolicibacteria, are not a permanent constituent of the microbiome, but because they have been regularly encountered in the diet and the environment, there is evidence for their evolutionary adaptedness (Rook, 2010). Interestingly, akin to the bacteria which make up the gut and skin microbiota, researchers have now identified communities of several of these operational mycobacterial taxonomic units in the oral cavity of healthy individuals (Macovei et al., 2015). This is presumably a reflection of the significant exposure to environmental NTB mycobacteria by this route. The extent to which NTB mycobacteria such as mycolicibacteria hold promise, like probiotics, for influencing human well‐being is the subject of ongoing research. Nevertheless, “the Old Friends hypothesis” makes a case for their benefit to human health as revealed by the drastic reduction of exposure to saprophytic environmental NTB mycobacteria in modern living conditions (Flandroy et al., 2018; Lowry et al., 2016; Rook et al., 2004, 2013).

Until recently, the assumption has been that probiotics should be viable to exert positive effects. Instead, there is now increasing evidence to show that nonviable probiotics maintain their health‐promoting benefits and a new term “postbiotic” has been coined to indicate preparations of inanimate microorganisms and/or their components that confer a health benefit to the host (Aguilar‐Toalá et al., 2018; Barros et al., 2020; Seminen et al., 2021; Taverniti & Guglielmetti, 2011). From a commercial standpoint, the use of nonviable bacteria has several advantages, including easing the challenges associated with product storage to maintain viability, reduction of safety concerns arising from horizontal virulence gene transfer from pathogenic bacteria, and the ability to deliver exact numbers of microorganisms per dose. In light of these issues, nonviable bacteria are now under consideration as novel food ingredients. In this report, we present evidence for the safety of heat‐killed *Mycolicibacterium aurum* Aogashima as a novel food ingredient. This is an environmental saprophytic organism which may not have the documented history of safe use that food‐associated probiotics have, but nonetheless, is likely to have been evolutionarily present in the diet, through exposure to untreated and even treated water supplies. The safety of this novel food was determined using the decision tree approach developed by Pariza and colleagues which relies on assessment of lack of allergenicity risk, confirmation that resistance to various antimicrobials is intrinsic and nontransmissible and that no harmful effects are detected in standard toxicology testing (Pariza et al., 2015). Our data support the conclusion that heat‐killed *M. aurum* Aogashima is safe as a novel food ingredient.

## 2 MATERIALS AND METHODS

### 2.1 Manufacture

*M. aurum* Aogashima has been deposited at the DSMZ (Deutsche Sammlung von Mikroorganismen und Zellkulturen GmbH, Germany) under the accession number DSM 33539. *M*. *aurum* Aogashima is manufactured following Good Manufacturing Practice (GMP) and Good Laboratory Practice (GLP) principles. The organism is grown in a bioreactor of either five or twenty‐five liters. Once an appropriate biomass is reached, the bacteria are recovered by centrifugation and resuspension in water, before heat inactivation at 121ºC for ≥20min. The resulting *M. aurum* Aogashima biomass is then further diluted with water and stored prior to use.

### 2.2 Safety evaluation process

**FIGURE 1:**
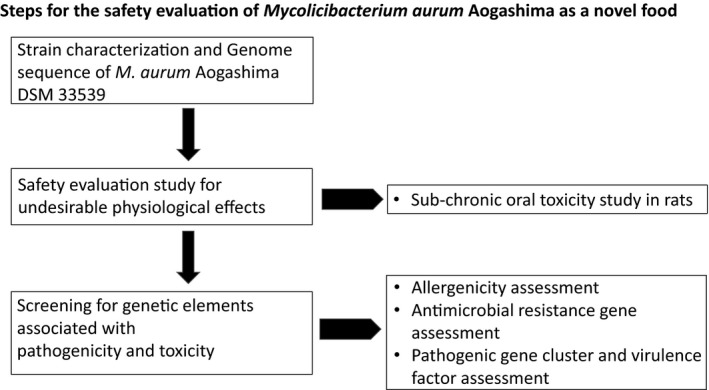
Flow chart describing the steps of the safety evaluation process based on Pariza et al., (2015)

The safety of *M. aurum* Aogashima was assessed based on the decision tree approach developed by Pariza and colleagues (2015). A flow chart describing the steps is depicted in Figure 1.

### 2.3 Subchronic oral toxicity study

The safety of heat‐killed *M. aurum* Aogashima was investigated in a 90‐day toxicity study in rats. Doses tested were selected on the basis of a 14‐day oral dose range finding study. Studies were GLP compliant, performed at Sequani Ltd (UK) according to United Kingdom GLP Regulations 1999, SI 1999 No. 3,106, as amended by SI 2004 No. 994, in accordance with the Organization for Economic Cooperation and Development (OECD) Guidelines (OECD, 1998). Briefly, 6‐ to 8‐week‐old male (*n* = 40) and female (*n* = 40) Crl:WI(Han) rats (Charles River, UK) were divided in groups of 10 males and 10 females and were dosed at 0 (vehicle control), 20, 200, or 2000 *μ*g/kg/day of heat‐killed *M. aurum* Aogashima, once daily by oral gavage, at a dose volume of 1 mL/kg body weight for at least 90 days, until the day before necropsy. Animals were housed in groups of 5, by sex and provided food and water ad libitum.

Animals were examined twice daily for mortality and morbidity. Any clinical signs of toxicity or changes in behavior or appearance were checked for daily. Body weights and food intake were recorded weekly until necropsy. Blood samples were taken for clinical pathology during week 13 according to OECD guidelines (1998). Hematological parameters investigated included changes in immune cell population counts. Blood chemistry parameters measured included markers associated with liver or kidney cellular toxicity, such as alanine and aspartate aminotransferase (ALT and AST, respectively), alkaline phosphatase (AP), urea, and creatinine. Animals were also subjected to a functional observational battery consisting of standard arena observations at predose and once weekly, together with an assessment at week 13 which included grip strength, motor activity, and sensorimotor responses to visual, acoustic, and proprioceptive stimuli according to OECD test guidelines (OECD, 1998). At the end of the treatment period, all animals were subjected to a gross necropsy, internal organs were weighed, and organ tissues from the control and high dose animals were examined microscopically.

### 2.4 Genome sequencing and analysis

DNA was extracted from a culture of *M. aurum* Aogashima as described in Amaro et al., (2008). Genome sequencing was performed using an Illumina MiSeq instrument, as previously described (Sangal et al., 2015). The genomes were assembled into contigs using SPAdes 3.9.0 with a kmer length of 127 and subsequently annotated using the Rapid Annotation of microbial genomes Subsystems Technology (RAST) server (Aziz et al., 2008; Bankevich et al., 2012).

### 2.5 Allergenicity assessment

Allergenicity potential of *M. aurum* Aogashima was assessed by AllerCatPro (https://allercatpro.bii.a‐star.edu.sg/), the most up‐to‐date database, comprising 4,180 unique allergenic protein sequences (Maurer‐Stroh et al., 2019). Briefly, linear sequences in the genome of *M. aurum* Aogashima were first compared to the allergen database to identify sequence windows of 80 residues with at least 35% of identity with proteins known to be allergenic as defined by FAO & WHO (2001). The amino acid sequences of *M. aurum* Aogashima genome were obtained after translation of nucleotide sequences using Prodigal software v2.6.3. The sequences with an identity above this threshold were then 3D‐modeled, and a B‐cell epitope‐like 3D surface was calculated and compared. Proteins with epitopes presenting an identity level of above 35% were considered allergens as outlined in Maurer‐Stroh et al., (2019).

### 2.6 Antimicrobial resistance gene assessment

The presence of genes coding for antibiotic resistance (AMR) was assessed in the genome of *M. aurum* Aogashima. The whole genomic sequence was compared against ResFinder databases and the Comprehensive Antibiotic Resistance Database (CARD) (Alcock et al., 2020; Zankari et al., 2012). Briefly, in silico genome analysis for the AMR genes was carried out by ResFinder 3.2 webserver which encompasses 15‐drug classes in its database: aminoglycoside, beta‐lactam, colistin, a fluoroquinolone, fosfomycin, fusidic acid, glycopeptide, macrolide‐lincosamide‐streptogramin B, nitroimidazole, oxazolidinone, phenicol, rifampicin, sulphonamide, tetracycline, and trimethoprim (Zankari et al., 2012). The percent identity and perfect alignment were set at 70% and 60%, respectively. The minimum length or the number of nucleotides that must overlap a resistant gene to count as a hit was set at the default of 60%.

The genome sequence of strain *M. aurum* Aogashima was interrogated for the presence of AMR genes based on CARD and using Resistance Gene Identifier (RGI) software for resistome analysis and prediction (Alcock et al., 2020). Each predicted AMR gene was manually mapped and annotated using the SEED and the RAST server (Aziz et al., 2012). Protein domains of AMR genes were confirmed after comparison with those available in the Conserved Domains Database (CDD) of NCBI (Marchler‐Bauer et al., 2015). Any hits were reported and analyzed.

### 2.7 Pathogenic gene clusters and virulence factors assessment

The draft genome sequence of *M. aurum* Aogashima was screened for pathogenic island and virulence factors using the Virulence Factor database (VFDB) (Liu et al., 2019). Experimentally validated virulence factors of major medically important bacterial pathogens belonging to 24 genera were considered. In addition, predicted coding sequences were identified using the GLIMMER3 system (system for finding genes in microbial DNA) prior to using the VFanalyzer tool (Virulence Factor analyzer tool). Lastly, blastp and Conserved Domain tools of NCBI were used to identify the virulence factors associated amino acid sequences of *M. aurum* and determine their functional similarity with those of *Mycobacterium tuberculosis* H37Rv. The established threshold of 60% for functional protein similarity was adopted (Addou et al., 2009).

### 2.8 Statistical Analysis

In vivo data were analyzed using Graph Pad Prism to give group mean values and standard error. Where appropriate and within each sex, one‐way ANOVA followed by Sidak's multiple comparisons test was used to determine statistical differences upon comparison of groups receiving different doses of heat‐killed *M. aurum* Aogashima versus the control group.

## 3 RESULTS

### 3.1 Subchronic oral toxicity study

**FIGURE 2:**
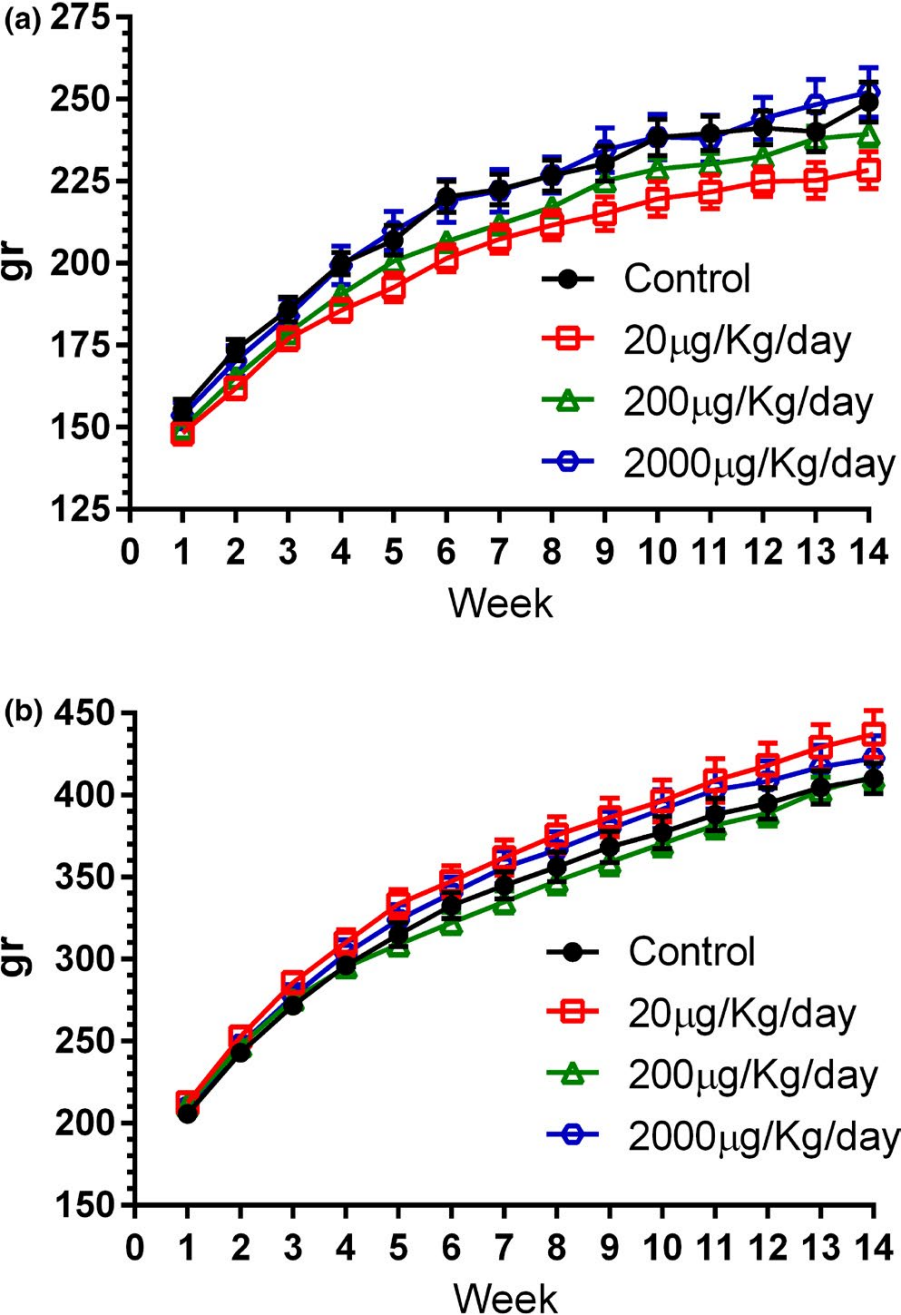
Comparison of body weight (gr) in female (A) and male (B) rats receiving vehicle control or 20, 200, and 2000 *μ*g/kg BW/day (10 males and 10 females per group) *M. aurum* Aogashima for 90 days. There was no treatment‐related effect on body weight or overall body weight gain. Data are shown as mean ± *SEM*

A 14‐day oral dose range finding study in Crl:WI(Han) rats was performed to determine doses to be tested further in a 90‐day oral toxicity study. This dose range study showed that administration of both 200 and 2000*μ*g/kg/day was well tolerated and 2000 *μ*g/kg/day was selected as the highest dose level in a subchronic oral toxicity study. Eighty Crl:WI(Han) rats (40 males and 40 females) were allocated into different dose groups receiving heat‐killed *M. aurum* Aogashima orally at 0 (vehicle control), 20, 200, and 2000 *μ*g/kg/day  (10 males and 10 females per group) for 90 days. Daily visual examinations from the start of treatment showed no deaths, no treatment‐related clinical signs of morbidity, toxicity, nor changes in behavior. Weekly measurements of body weight and food intake revealed no significant differences between groups. All groups gained a similar amount of weight (Figure 2) and ate a similar amount of food (data not shown) when compared to control groups. Animals showed no evidence for treatment‐related neurotoxicity based on functional observation battery assessments. Indeed, there were no effects on functional arena observations or on grip strength or motor activity and sensorimotor responses to visual, acoustic, and proprioceptive stimuli (data not shown). At the end of the treatment period, all animals were subjected to a gross necropsy where organs were weighed and examined macroscopically. We detected no effect on organ weights in the male groups regardless of treatment. In the female group, we observed only a significant decrease in the liver weight and only in the group receiving 20 *μ*g/kg/day (Table 1). Nevertheless, there were no differences detected in the percentages of organ weight in relation to body weight in the 20 *μ*g/kg/day dose group compared to control group (3.32 ± .067 versus 3.39 ±  .037%, respectively). Moreover, no abnormal macroscopic observations were reported. Hence, because the observed difference in the weights of livers was not dose dependent, did not translate into changes in organ weight percentages or in macroscopic changes, and was found not to be associated with increases in liver enzymes indicative of hepatocellular toxicity such as AST, ALT, and AP (Table 2), it was deemed not to have biological significance. Furthermore, there were no macroscopic or when performed microscopic abnormalities related to oral consumption of heat‐killed *M. aurum* Aogashima in any organs of any groups.

**TABLE 1:** Effects of feeding different doses of *M. aurum* Aogashima on selected tissue organs. Data are shown as mean (*n* = 10) ± *SEM*. No significant differences were observed compared to relevant vehicle control group in the males; * indicates significant differences in the female group

Group	Heart gr		Kidneys gr		Liver gr		Spleen gr		Thyroid mg		Adrenal mg	
	♀	♂	♀	♂	♀	♂	♀	♂	♀	♂	♀	♂
0 *μ*g/Kg/day Vehicle Control	.85 ± .04	1.15 ± .04	1.64 ± .05	2.41 ± .05	8.47 ± .28	13.58 ± .44	.57 ± .03	.65 ± .02	18.80 ± 1.11	22.2 ± .44	73 ± 4.33	60 ± 3
20 *μ*g/Kg/day	.77 ± .03	1.26 ± .04	1.57 ± .05	2.51 ± .09	7.54 ± .26 ^*^p =.039	13.79 ± .43	.56 ± .03	.68 ± .03	16.70 ± 1.32	23.1 ± .82	80 ± 3.67	57 ± 1.67
200 *μ*g/Kg/day	.82 ± .02	1.17 ± .04	1.61 ± .05	2.46 ± .09	8.02 ± .22	13.49 ± .45	.56 ± .02	.65 ± .02	17.90 ± .88	21.70 ± 1.11	75 ± 2.67	57 ± 3.33
2000 *μ*g/Kg/day	.86 ± .02	1.22 ± .04	1.66 ± .05	2.56 ± .07	8.42 ± .26	13.82 ± .54	.54 ± .04	.69 ± .02	17.10 ± 1.42	22.20 ± 1.39	73 ± 2.33	59 ± 3.33

**TABLE 2:** Effects of feeding different doses of *M. aurum* Aogashima on selected blood chemistry parameters. Data are shown as mean(*n* = 10) ± *SEM*. No significant differences were observed compared to relevant vehicle control group

Group	Alanine Aminotrasferase U/l		Aspartate Aminotransferase U/l		Alkaline Phosphatase U/l		Urea mg/dl		Creatine mg/dl	
	♀	♂	♀	♂	♀	♂	♀	♂	♀	♂
0 *μ*g/Kg/day Vehicle Control	29.40 ± 2.70	36.90 ± 2.08	71.70 ± 11.02	61.50 ± 2.69	49.90 ± 4.09	114.30 ± 8.31	34.77 ± 1.32	37.30 ± 1.61	.26 ± .01	.26 ± .01
20 *μ*g/Kg/day	28.20 ± 1.30	33.50 ± 2.28	64.30 ± 3.45	60.10 ± 2.50	57.80 ± 4.52	105.60 ± 6.06	34.38 ± .96	37.55 ± 1.16	.25 ± .004	.25 ± .01
200 *μ*g/Kg/day	28.10 ± .81	31.80 ± .62	59.00 ± 2.83	53.10 ± 1.24	51.60 ± 4.53	105.30 ± 7.89	34.5 ± 1.27	35.55 ± 1.1	.26 ± .01	.25 ± .01
2000 *μ*g/Kg/day	29.10 ± 1.37	31.80 ± 1.69	62.30 ± 5.77	60.80 ± 3.59	53.50 ± 4.26	104.30 ± 4.69	37.53 ± 2.03	34.63 ± .73	.26 ± .003	.22 ± .01

Prior to necropsy, blood samples were taken for clinical pathology analysis at week 13. Statistical analysis revealed no significant differences in the groups on any of the blood chemistry parameters which are routinely considered to be broadly indicative of hepatocellular or renal toxicity (Table 2). Among all blood chemistry parameters measured, we observed an out of “normal” range significant difference (*p <.05) in glucose levels compared to control animals. Levels (mg/dl) in control male rats (191.8 ± 7) were comparable to level in rats receiving 20 and 2000 *μ*g/Kg/day (193.7 ± 6.5 and 196.3 ± 7, respectively) but significantly lower than levels in rats receiving 200 *μ*g/Kg/day (221.1 ± 8.6). Similarly, levels in control female rats (163.7 ± 4.5) were comparable to levels in rats receiving 20 *μ*g/Kg/day (179.8 ± 6.7) but significantly lower than those in rat receiving 200 and 2000 *μ*g/Kg/day (191.7 ± 6 and 190.3 ± 5.2, respectively). As glucose levels were already higher than normal (106–184 in males and 89–163 mg/dl in females) in the control groups and rats were not fasted overnight prior to blood sampling, we consider these may be normal biological variations due to food consumption and circadian rhythms.

Hematological parameters were also assessed. There were no statistically significant changes in total white blood cell and immune cell population specific counts in the female groups, aside from a significant decrease in monocytes in the group receiving 200 *μ*g/Kg/day  (Table 3). However, when cell population percentages were calculated, no significant differences were detected in any cell populations, including monocytes, regardless of dose received (Table 4). In males, we observed a significant decrease in white blood cell, neutrophils, and lymphocytes absolute counts only in the group receiving 200 *μ*g/Kg/day  (Table 3). However, we found no evidence for changes in the percentages of these populations or in any other immune cell populations (Table 4). Moreover, values remained within normal range (white blood cells 1.98–11.06 x 10^3^/*μ*l; neutrophils .33–1.98 10^3^/*μ*l). Due to the lack of a dose relationship, given no differences were detected in the highest dose groups (which received concentrations 10 times of those where differences were observed), and because there were no changes in overall immune cell population percentages, we consider these observations part of normal biological variation rather than any effect of oral consumption of *M. aurum* Aogashima. Hence, the reported data concluded no observed adverse effect level (NOAEL) at all doses tested including the highest dose tested of 2000*μ*g/Kg/day.

**TABLE 3:** Effects of feeding different doses of *M. aurum* Aogashima on selected hematological parameters. Data are shown as mean (*n* = 10) ± *SEM*. * indicates significant differences in the male and female group

Group	White Blood Cell 10^3^/*μ*l		Neutrophils 10^3^/*μ*l		Lymphocytes 10^3^/*μ*l		Monocytes 10^3^/*μ*l		Eosinophils 10^3^/*μ*l		Basophils 10^3^/*μ*l	
	♀	♂	♀	♂	♀	♂	♀	♂	♀	♂	♀	♂
0 *μ*g/Kg/day Vehicle Control	5.52 ± .47	7.79 ± .36	.63 ± .10	1.± .09	4.56 ± .36	6.45 ± .29	.16 ± .02	.14 ± .02	.13 ± .02	14 ± .01	.01 ± .0	.03 ± .0
20 *μ*g/Kg/day	5.45 ± .44	6.77 ± .26	.70 ± .04	.93 ± .05	4.45 ± .40	5.46 ± .25	.13 ± .01	.15 ± .03	.12 ± .02	.18 ± .03	.01 ± .0	.02 ± .0
200 *μ*g/Kg/day	4.39 ± .33	6.12 ± .39 *p =.009	.58 ± .07	.76 ± .05 *p =.045	3.57 ± .28	5.07 ± .4 *p =.026	.10 ± .01 *p =.016	.13 ± .01	.11 ± .01	.11 ± .01	.01 ± .0	.02 ± .0
2000 *μ*g/Kg/day	4.9 ± .32	6.83 ± .34	.66 ± .08	.87 ± .05	3.98 ± .27	5.66 ± .35	.13 ± .02	.12 ± .01	.1 ± .01	.13 ± .01	.01 ± .0	.02 ± .0

**TABLE 4:** Effects of feeding different doses of *M. aurum* Aogashima on selected hematological parameters. Data are shown as mean (*n* = 10) ± *SEM*. No significant differences were observed compared to relevant vehicle control group

Group	% Neutrophils		% Lymphocytes		% Monocytes		% Eosinophils		% Basophils	
	♀	♂	♀	♂	♀	♂	♀	♂	♀	♂
0 *μ*g/Kg/day Vehicle Control	11.08 ± .97	12.79 ± .90	82.87 ± 1.14	82.81 ± .9	2.95 ± .43	1.72 ± .23	2.30 ± .22	1.79 ± .13	.19 ± .03	.33 ± .05
20 *μ*g/Kg/day	13.28 ± .87	13.82 ± .88	81.30 ± 1.08	80.6 ± 1.32	2.46 ± .19	2.25 ± .41	2.28 ± .33	2.58 ± .39	.18 ± .03	.26 ± .03
200 *μ*g/Kg/day	13.22 ± 1.31	12.89 ± 1.21	81.29 ± 1.34	82.31 ± 1.31	2.34 ± .15	2.08 ± .21	2.60 ± .18	1.87 ± .20	.12 ± .03	.29 ± .03
2000 *μ*g/Kg/day	13.45 ± 1.38	13.02 ± 1.12	81.23 ± 1.4	82.49 ± 1.33	2.60 ± .30	1.79 ± .15	2.04 ± .20	1.88 ± .18	.16 ± .03	.34 ± .07

### 3.2 Allergenicity

The allergenicity potential of *M. aurum* Aogashima was assessed by an innovative 3D‐modeling‐based analysis, using the AllerCatPro database. Only fifteen potentially allergenic protein sequences were detected with linear sequence window identity above the thresholds of 35% (Table 5). Most of the detected amino acids sequences in the genome of *M. aurum* Aogashima corresponded to allergenic proteins previously found in fungi (56%), while 26%, 15%, and 3% of the predicted proteins belong to foods, arthropods, and mammals (just one sequence), respectively. None of these proteins showed a 3D epitope identity, and therefore, we concluded that there was no evidence for allergenicity following consumption of heat‐killed *M. aurum* Aogashima (Table 5).

**TABLE 5:** In silico genome analysis for allergenic proteins of *M. aurum* Aogashima using AllerCatPro server

Known allergen hit name		% identity linear 88aa window	% identity 3D epitope
Alcohol Dehydrogenase	*Candida albicans*	41.1 – 67.5	‐
ALF_CANAL fructose‐bisphosphate aldolase	*Candida albicans*	55	‐
Aldehyde dehydrogenase Cla h 10	*Cladosporium herbarum*	48.8	‐
Mannitol dehydrogenase Cla h8	*Cladosporium herbarum*	37.5 – 38.2	‐
NADP‐dependent mannitol dehydrogenase	*Alternaria alternata*	37.7 – 40.8	‐
Probable beta‐glucosidase ARB_05654 BGLA_ARTBC	*Arthroderma benhamiae*	51.2	‐
THIO_COPCM Thiredoxin	*Coprinus comatus*	44.9	‐
Asp f IAO	*Aspergillus fumigatus*	43	‐
Asp f FDH	*Aspergillus fumigatus*	40	‐
Pen C	*Penicillum citrinum*	58.8	‐
Seed maturation‐like protein precursor	*Sesamum*	37.5 – 45	‐
Tri a 34.0101 (glyceraldehyde−3‐phosphate dehydrogenase)	*Triticum aestivum*	71.2	‐
Aldehyde dehydrogenase‐like protein Tyrophagus	*Tyrophagus putrescentiae*	56.2	‐
*Cul n 8*	*Culicoides nubeculosus*	41.1	‐
Cyclophilin, CyP	Mammals	57.5	‐

### 3.3 Antimicrobial resistance gene assessment

We found no hits between the genome of *M. aurum* Aogashima and the AMR genes included in ResFinder databases. Instead, 3 hits above 70% identity: *rbp*A (RbpA bacterial RNA polymerase‐binding protein), *mtr*A (resistance‐nodulation‐cell division antibiotic efflux pump), and *mur*A transferase (*Mycobacterium tuberculosis* intrinsic *mur*A conferring resistance to fosfomycin) were reported using CARD webserver. These genes confer resistance to rifampicin, penam, and fosfomycin, respectively. However, as reported in Table 6, all hits were below the general reference value for gene homology (97%). Furthermore, these genes have been reported to be widely present in the *Mycobacteriaceae* and, therefore, not surprisingly also in the *M. aurum* type strain DSM 43999^T^ (Table 6).

**TABLE 6:** AMR genes detected in the genome sequence of *M. aurum* Aogashima and its relative *M. aurum* type strain DSM 43999^T^ with an identity value ≥70%

			% Identity matching Region		% length or reference sequence	
Genes AMR Gene Family	Drug Class	Resistance Mechanisms	*M. aurum* Aogashima	*M. aurum* Type strain	*M. aurum* Aogashima	*M. aurum* Type strain
*rbp*A (RbpA bacterial RNA polymerase‐binding protein)	Rifampicin	Antibiotic target protection	96.4	96.4	97.4	100
*mtr*A (resistance‐nodulation‐cell division antibiotic efflux pump)	Macrolide antibiotic, penam	Antibiotic efflux	96.9	96.9	100	97.4
*mur*A transferase (*Mycobacterium tuberculosis* intrinsic murA conferring resistance to fosfomycin)	Fosfomycin	Antibiotic target alterations	92.4	92.4	98.5	98.5

### 3.4 Pathogenic gene clusters and virulence factors assessment

The whole genome sequence of *M. aurum* Aogashima was screened to identify genetic element sequences that encode for virulence factors or protein toxins. We found no evidence for pathogenic islands. Screening of the genome of *M. aurum* Aogashima for all known virulence factors associated genes showed that most of the predicted genes were found in nonpathogenic or commensal bacteria and are involved in host interaction, survival, and maintenance of basic functions (Table 7). As shown in Table 7, several proteins with experimentally verified virulence factors were present in the genome of *M. aurum* Aogashima but their amino acid sequence similarity with that of the pathogenic *M. tuberculosis* is below the 60% cutoff value for functional homology (Table 7).

**TABLE 7:** Virulence factors associated genes detected in the genome sequences of *M. aurum* Aogashima and *M. tuberculosis* H37Rv

VFclass	Virulence factors	Related genes	*M. aurum* Aogashima	*M. tuberculosis* H37Rv	Similarity (%)	comments	e‐values
Amino acid and purine metabolism	Glutamine synthesis	*gln*A1	orf04741	Rv2220	84.3	<95%	0
	Leucine synthesis	*leu*D	orf04409	Rv2987c	84.2	<95%	6E−127
	Lysine synthesis	*lys*A	orf04608	Rv1293	79.3	<95%	0
	Nitrate/nitrite transporter	*nar*K2	orf00718	Rv1737c	25.6	<60%	1E−14
Catabolism of cholesterol	Cyp125	*cyp*125	orf01099; orf01566; orf04038; orf04661	Rv3545c	54.4	<95%	4E−172
	FadE28	*fad*E28	orf04037	Rv3544c	68.5	<95%	1E−164
	FadE29	*fad*E29	orf04036	Rv3543c	81.6	<95%	0
Cell surface components	Carboxylesterase	*cae*A	orf04747; orf04749	Rv2224c	53.6	<60%	3E−169
	Exported repetitive protein	*erp*	orf04235	Rv3810	49.6	<60%	6E−58
		*fad*23	orf01422	Rv1185c	60.2	<95%	0
		*fad*E5	orf01415; orf05079	Rv0244c	82.7 and 66.1	<95%	0
		*gtf*1	orf01592	Rv1526c	48.8	<95%	1.3
		*gtf* 2	orf01265; orf01577	Rv1524	58.2 and 53.8	<95%	9E−162
		*mmp*L10	orf01421	Rv1183	57.2	<95%	0
		*mmp*S4	orf00497; orf04089	Rv0451c	47.5	<95%	3E−47
		*mp*s1	orf02234	Rv0101	49.3	<95%	0
		*pap*A3	orf01420	Rv1182	54.5	<95%	0
		*rml*A	orf01604; orf05016	Rv0334	46.7	<95%	4E−161
	Heparin‐binding hemagglutinin	*hbh*A	orf03209	Rv0475	67.9	>60%	5E−64
	Lipoprotein	*lpr*G	orf05150	Rv1411c	52.3	<95	4E−78
	Methyltransferase	*mma*A4	orf00819	Rv0642c	68.1	<95	1.5
	MymA operon	*adh*D	orf03671; orf04049; orf05070; orf05579	Rv3086	34.1	<60%	.016
		*fad*D13	orf01899	Rv3089	36.4	<60%	2E−95
		*mym*A	orf05192	Rv3083	50.0	<60%	why not 95%
		*ddr*A	orf00094; orf01270; orf02392	Rv2936	46.4, 51.5, 67.8	<60%	1E−79
		*ddr*B	orf02393	Rv2937	43.1	<60%	1.00E−76
		*drr*C	orf02394	Rv2938	46.7	<60%	2E−90
		*fad*D26	orf02385; orf02387	Rv2930	60.2, 55.1	<60%	0
		*fad*D28	orf01272; orf01571	Rv2941	55.3, 63.4	<60%	0
		*pps*A	orf02386	Rv2931	56.0	<60%	0
		*pps*B	orf02388; orf02389	Rv2932	54.0, 55.1	<60%	0
		*pps*D	orf02390	Rv2934	58.8	<60%	0
		*pps*E	orf02391	Rv2935	34,7	<60%	1E−125
	Proximal cyclopropane synthase of alpha mycolates	*pca*A	orf00241; orf00242; orf00820; orf01613	Rv0470c	60.0, 56.3, 68.9, 96.2	<95	2E−123
	Sulfolipid−1 biosynthesis and transport	*mmp*L8	orf01266	Rv3823c	50.4	<95	0
		*pap*A1	orf01262	Rv3824c	51.0	<95	2E−170
		*lpq*Y	orf03429	Rv1235	28.3	<60%	2E−53
		*sug*A	orf03428	Rv1236	53.0	<60%	3E−91
		*sug*B	orf03427	Rv1237	57.1	<60%	3E−109
		*sug*C	orf03426	Rv1238	53.9	<60%	8E−142
Copper uptake	Copper exporter	*ctp*V	orf03576	Rv0969	54.3	<60%	0
		*fad*D33	orf02653	Rv1345	61.7	<95%	0
		*Mbt*A	orf00510	Rv2384	64.3	<95%	0
		*Mbt*B	orf00509	Rv2383c	60.9	<95%	1E−23
		*Mbt*C	orf00507	Rv2382c	75.0	<95%	0
		*Mbt*D	orf00506	Rv2381c	51.6	<95%	0
		*Mbt*E	orf00505	Rv2380c	67.4	<95%	0
		*Mbt*F	orf00504	Rv2379c	55.6	<95%	0
		*Mbt*G	orf00503	Rv2378c	76.9	<95%	.16
		*Mbt*H	orf00502; orf01576	Rv2377c	73.5	<95%	6E−41
		*Mbt*J	orf04363	Rv2385	60.6	<95%	4E−131
		*Mbt*K	orf05262	Rv1347c	62.7	<95%	4E−88
	Pantothenate synthesis	*Pan*C	orf05603	Rv3602c	70.5	<95%	6E−138
		PanD	orf00584	Rv3601c	63.1	<95%	5E−56
Antiapoptosis factor	NuoG	*Nuo*G	orf02508	Rv3151	73.1	>60%	0
Mammalian cell entry (mce) operons	Mce1	*mce*1A	orf01133	Rv0169	53.3	‐	1E−150
		*mce* 1B	‐	Rv0170	‐		‐
		*mce* 1C	‐	Rv0171	‐		‐
		*mce*1D	orf04143	Rv0172	67.6		0
		*mce*1E	‐	Rv0173	‐		‐
		*mce*1F	orf04141	Rv0174	68.1		0
	Mce2	*mce*2A	orf04716	Rv0589	66.5	‐	0
		*mce*2B	orf04717	Rv0590	72.4		3E−129
		*mce*2C	‐	Rv0591	‐		‐
		*mce*2D	‐	Rv0592	‐		‐
		*mce*2E	orf04142	Rv0593	67.8		0
		*mce*2F	‐	Rv0594	‐		‐
	Mce3	*mce*3A	orf03117	Rv1966	65.3	>60%	6E−180
		*mce*3B	orf03118; orf05701	Rv1967	68.1	>60%	1E−163
		*mce*3C	orf03119	Rv1968	61.5	>60%	4E−164
		*mce*3D	orf03120	Rv1969	63.6	>60%	2E−04
		*mce*3E	orf03121; orf04341	Rv1970		>60%	0
		*mce*3F	orf03122; orf04340	Rv1971	58.9	>60%	1E−179
	Mce4	*mce*4A	orf01047; orf01461	Rv3499c	66.7	>60%	0
		*mce*4B	orf01048; orf01460	Rv3498c	69.1	>60%	1E−168
		*mce*4C	orf01049; orf01459	Rv3497c	67.7	>60%	2E−171
		*mce*4D	orf01050; orf01458	Rv3496c	63.8	>60%	0
		*mce*4E	orf01051; orf01457	Rv3495c	64.4	>60%	6E−179
		*mce*4F	orf01052; orf01456	Rv3494c	69.6	>60%	0
Phagosome arresting	Nucleoside diphosphate kinase	*ndk*	orf05384	Rv2445c	80.7	>60%	2E−81
	PE family protein	PE_PGRS30	‐	Rv1651c			
	Tyrosine phosphatase	*ptp*A	orf00358	Rv2234	70.0	>60%	3E−82
Secreted proteins	19‐kD protein	*lpq*H	orf04515	Rv3763	60.1	<95%	3E−59
	Alpha‐crystallin	*hsp*X	orf00236; orf05393	Rv2031c	39.0	<60%	3E−18
	Antigen 85 complex	*eis*	orf02486	Rv3804c	70.6	>60%	4E−150
		*fbp*B	‐	Rv1886c			
		*fbp*C	orf01653; orf02135; orf04229; orf04918	Rv0129c	76.1	>60%	5E−175
	Enhanced intracellular survival protein	*eis*	orf05310	Rv2416c	56.1	<60%	4E−150
	ESX−1 (T7SS)	*PE*35	orf05503	Rv3872	47.7	<60%	3E−26
		*PPE*68	orf05504	Rv3873	41.5	<60%	7E−63
		*ecc*A1	orf05499	Rv3868	76.7	>60%	0
		*ecc*B1	orf05500	Rv3869	64.2	>60%	0
		*ecc*Ca1	orf05501	Rv3870	80.0	>60%	0
		*ecc*Cb1	orf05502	Rv3871	73.3	>60%	0
		*ecc*D1	orf05508	Rv3877	66.6	>60%	0
		*ecc*E1	orf04675	Rv3882c	67.5	>60%	0
		*esp*I	orf03155; orf05507	Rv3876	34.9	<60%	6E−49
		*esp*J	orf05509	Rv3878	32.2	<60%	2E−12
		*esp*K	orf05512	Rv3879c	55.0	<60%	5E−88
		*esp*L	orf05699	Rv3880c	53.8	<60%	4E−31
		*esp*R	orf05183	Rv3849	80.1	>60%	4E−80
		*esx*A	orf05506	Rv3875	54.4	<60%	5E−31
		*esx*B	orf05505	Rv3874	43.4	<60%	2E−19
		*myc*P1	orf04676	Rv3883c	71.8	>60%	0
	ESX−3 (T7SS)	*PE*5	orf01528	Rv0285	69.8	>60%	7E−31
		*PP*E4	orf01529	Rv0286	58.4	<60%	4E−79
		*ecc*A3	orf01525	Rv0282	73.1	>60%	0
		*ecc*B3	orf01526	Rv0283	63.0	>60%	0
		*ecc*C3	orf01527	Rv0284	74.7	>60%	0
		*ecc*D3	orf01533	Rv0290	62.8	>60%	9E−176
		*ecc*E3	orf01535	Rv0292	50.8	<60%	1E−80
		*esp*G3	orf01532	Rv0289	55.3	<60%	3E−115
		*esx*G	orf01530	Rv0287	76.6	>60%	7E−40
		*esx*H	orf01531	Rv0288	72.6	>60%	6E−52
		*myc*P3	orf01534	Rv0291	60.9	>60%	0
	ESX−4 (T7SS)	*ecc*B4	orf00970	Rv3450c	48.5	<60%	5E−125
		*ecc*C4	orf00973	Rv3447c	51.8	<60%	0
		*ecc*D4	orf00972	Rv3448	30.0	<60%	4E−16
		*esx*T	orf00976	Rv3444c	64.8	>60%	3E−44
		*esx*U	orf00975	Rv3445c	64.3	>60%	3E−46
		*myc*P4	orf00971	Rv3449	54.4	<60%	2E−139
		*cyp*143	orf00285	Rv1785c	60.0	borderline of functional identity	4E−168
	Catalase‐peroxidase	*kat*G	orf02989; orf04948	Rv1908c	65.5	>60%	0
	Cu	*sod*C	orf02589; orf04790	Rv0432	33.3	<60%	2.4

## 4 DISCUSSION

Humans have evolved in a microbial world. The resulting evolutionary adaptedness is based on microbes’ colonization of human skin and mucosal surfaces as well as regular microbial contact in the air, surfaces, and in food and beverages (Rook, 2010). Until very recently, diet provided the most exposure through raw, minimally processed or fermented foods and beverages and through untreated water. A link between consumption of live microbes—such as those found in fermented food—and health has been reported in both intervention and associative studies as well as randomized controlled trials (Marco et al., 2017; Sanlier et al., 2019). As health evidence is mounting, there have been calls to include recommendations for the consumption of microbes in dietary guidelines, akin to the ones related to dietary fibers (Marco et al., 2020). For these reasons, microbes have gained increasing interest as potential novel food ingredients.

Numerous microbes are currently being investigated for their safety as novel food ingredients and for their potential benefit to human health. These include novel probiotics such as *Clostridium butyricum* CBM588, as well as postbiotics, defined as inanimate bacterial preparations which confer health benefit to the host. The latter would include *Yarrowia lipolyticus*, *Mycobacterium setense* strain *Manresensis,* and pasteurized *Akkermansia municiphila* among others (Aguilar‐Toalá et al., 2018; Akter et al., 2020; Barros et al., 2020; Cani & de Vos, 2017; EFSA Panel on Nutrition et al., 2019a; 2019b; Kanai & Mikani, 2015; Salminen et al., 2021; Taverniti & Guglielmetti, 2011). The food use of dead microbes has several advantages compared to live organisms: the difficulties of ensuring cell viability at the levels reported in the product description and for the duration of their shelf‐life are avoided, for example. Similarly, using heat‐killed organisms limits concerns arising from use of these products in vulnerable groups such as the very young and immunosuppressed individuals and allows for more widespread use (Piqué et al., 2019). Interestingly, the organism under study in this report, heat‐killed *M. aurum* Aogashima, may fall within the definition of postbiotic, should a health benefit for this preparation be shown in separately presented studies. The purpose of the work described here, however, is solely to present and assess the evidence for the safe use of heat‐killed *M. aurum* Aogashima as a novel food ingredient.

This environmental saprophytic organism is likely to have been long present in the diet as a harmless water contaminant (Falkinham et al., 2001; Le Dantec et al., 2002a, 2002b; Vaerewijck et al., 2005). Safety of heat‐killed *M. aurum* Aogashima as a novel food ingredient was assessed according to the decision tree approach developed by Pariza and colleagues (2015). The interest in expanding the number of microbes being considered as novel food, beyond the current standardized cultures and probiotics supplements, has driven a new approach to assess safety. This new framework is also pertinent to those cultures that are perceived to lack an established history of safe use for their intended application. We provide evidence that the genome of *M. aurum* Aogashima is free of (1) genetic elements associated with pathogenicity or toxigenicity, (2) transferable antibiotic resistance gene DNA, and (3) genes coding for antibiotics used in human or veterinary medicine. Moreover, our evidence shows that (4) the no observed adverse effect level (NOAEL) was the highest dose tested, 2000 *μ*g/kg BW/day.

Genetic elements associated with pathogenicity or toxigenicity were investigated by extensive in silico analysis and showed no evidence of pathogen‐specific virulence factors in *M. aurum* Aogashima. Indeed, the virulence factors associated genes identified were common to both pathogenic and nonpathogenic and commensal bacteria and associated with highly conserved functions such as amino acid and purine metabolism and the catabolism of cholesterol and were not located on pathogenic island (Niu et al., 2013). Hence, these highly conserved coding sequences are not considered appropriate markers of pathogenicity of *M. aurum* Aogashima. In this context, the presence of secreted protein associated genes (e.g., *fbp*A) is expected because they play a fundamental role in cell envelope maintenance (Belisle et al., 1997). The same can be said with respect to the genes *ptpA* and *ptpB* which are widely distributed among pathogenic and nonpathogenic mycobacterial species and also found in the genomes of other prokaryotes, including *Lactobacillus spp*. (Altermann et al., 2005; Boekhorst et al., 2006). It should also be noted that following comprehensive phylogenomics and comparative genomic analysis on 150 genomes of *Mycobacterium spp,* Gupta and his colleagues have reclassified the *Mycobacterium* genus into five distinct monophyletic groups (Gupta et al., 2018). As a result, what was once known as *Mycobacterium aurum* has been reclassified into the novel genus *Mycolicibacterium* (“*Fortuitum‐Vaccae*” clade) which is comprised of rapidly growing environmental species that are divergent from the clinical pathogenic *Mycobacterium* species. Hence, the absence of true virulence genes and pathogenic island in the genome sequence of *M. aurum* Aogashima are in line with its assignment to the species *M. aurum* known for its nonpathogenic trait (Risk group 1).

Antimicrobial resistance gene assessment was made by screening the genome using both ResFinder and CARD webservers to ensure coverage of all AMR determinants (i.e., acquired resistance genes, resistant mutations of housekeeping genes, efflux overexpression, etc.), drug targets, antibiotic molecules and drug classes, and the molecular mechanisms of resistance (Alcock et al., 2020; Zankari et al., 2012). We found no evidence for any resistance genes associated with the most common antimicrobial compounds of concern in food (namely, Ampicillin, Chloramphenicol, Kanamycin, Streptomycin; Erythromycin, Gentamycin, Tetracyclin, Vanomycin, and Lincomycin). We did, however, detect similarities with *rbpA*, *mtrA,* and *mur*A. While these genes are known to confer resistance to rifampicin, penam, and fosfomycin, respectively, their identity values were close to, but still below, the cutoff of 97% homology. Moreover, these genes are commonly present in mycobacteria as they are likely involved in essential cell functions (Maitra et al., 2019; Newell et al., 2006). Finally, there is no evidence for transferability. Hence, the absence of significant resistance genes in *M. aurum* Aogashima as well as the use of this organism as a heat‐killed preparation intended as novel food ingredient supports the conclusion that this is a safe product.

*M. aurum* Aogashima was also evaluated for allergenic potential using the traditional FAO/WHO issued guidelines as well as an innovative 3D‐modeling‐based analysis (AllerCatPro database). It was concluded that *M. aurum* Aogashima would not trigger any allergenic or hypersensitivity reactions in humans. Based on the low number of the predicted allergenic protein sequences detected in the genome by the traditional methodology, and the absence of 3D epitope similarity, it is highly probable that this organism does not produce any true allergenic proteins. Indeed, only fifteen protein sequences were deemed as potentially allergenic based on linear sequence window identity (80 residues) above the thresholds of 35% (traditional methodology). Of those, only two predicted allergenic proteins in *M. aurum* Aogashima related to food. None of those showed 3D epitope identity, strongly suggesting that the predicted protein sequence matches might be false positives.

Safety of heat‐killed *M. aurum* Aogashima was further assessed by toxicology testing, including a subchronic (90 day) oral challenge using male and female Crl:WI(Han) adult rats. All doses tested, including the highest doses of 2000 *μ*g/Kg/day, had no treatment‐related adverse effects. No relevant abnormalities between groups receiving *M. aurum* Aogashima and the control group were detected upon statistical analysis in a variety of parameters evaluated. Indeed, statistical differences were limited to differences in specific immune cell counts, but did not apply to differences in percentages of the same cell population. Furthermore, all cellular values remained well within the natural healthy range for adult rats (Giknis & Clifford, 2008). In the case of the reported glucose levels, we consider these may be normal biological variations due to continuous access to food and the effects of circadian rhythms (Kohsaka & Bass, 2007). For these reasons, and because of the absence of a dose relationship (given no differences were detected in the highest dose groups which received doses 10 times of those where differences were observed), we consider these differences part of normal biological variation rather than any effect of consumption of heat‐killed *M. aurum* Aogashima.

Based on the findings of the work and analysis described here, our conclusion is that the use of heat‐killed *M. aurum* Aogashima in food products is safe and that it is suitable for being evaluated as a novel food ingredient.

## 5 ETHICAL APPROVAL

All animal work performed at Sequani Ltd was conducted conforming to the UK legislation under the Animal (Scientific Procedures) Act 1986 (ASPA) Amendment Regulations (SI 2012/3039). Sequani Ltd is fully accredited by the Association for Assessment and Accreditation of Laboratory Animal Care International (AAALAC).

## CONFLICT OF INTEREST

TD is a senior executive and holds stock in Aurum Switzerland AG. IN has no conflict of interest to declare.

## AUTHOR CONTRIBUTION

**Imen Nouioui:** Conceptualization (supporting); Data curation (lead); Formal analysis (lead); Investigation (lead); Methodology (lead); Project administration (supporting); Resources (lead); Supervision (lead); Validation (equal); Visualization (supporting); Writing‐original draft (equal); Writing‐review & editing (equal). **Timothy Dye:** Conceptualization (lead); Formal analysis (supporting); Funding acquisition (lead); Project administration (lead); Supervision (supporting); Validation (equal); Visualization (lead); Writing‐original draft (equal); Writing‐review & editing (equal).

## Data Availability

The data that support the findings of this study are available from the corresponding author upon reasonable request.
